# Spatiotemporal dynamics of HIV-1 transmission in France (1999–2014) and impact of targeted prevention strategies

**DOI:** 10.1186/s12977-017-0339-4

**Published:** 2017-02-21

**Authors:** Antoine Chaillon, Asma Essat, Pierre Frange, Davey M. Smith, Constance Delaugerre, Francis Barin, Jade Ghosn, Gilles Pialoux, Olivier Robineau, Christine Rouzioux, Cécile Goujard, Laurence Meyer, Marie-Laure Chaix, on behalf the ANRS PRIMO Cohort Study

**Affiliations:** 1 University of California, San Diego, 9500 Gilman Drive, Stein Clinical Research Building #325, La Jolla, CA 92093-0697 USA; 2 INSERM CESP U1018, University Paris Sud, Hôpital Bicêtre, Assistance Publique-Hôpitaux de Paris (APHP), Le Kremlin-Bicêtre, France; 3 EA7327, Université Paris Descartes, Paris, France; 4 Laboratoire de Microbiologie Clinique, Hôpital Necker – Enfants Malades, APHP, Paris, France; 5 Veterans Affairs San Diego Healthcare System, San Diego, CA USA; 6 INSERM U941, Laboratoire de Virologie, Université Paris Diderot, Hôpital Saint-Louis, AP-HP, CNR VIH associé Primo infection, Paris, France; 7 INSERM U966 and National Reference Center for HIV, CHU Bretonneau and Université François Rabelais, Tours, France; 8 UF de Thérapeutique en Immuno-Infectiologie, Hôpital Hôtel Dieu, APHP, Paris, France; 9 Service des Maladies Infectieuses et Tropicales, Hôpital Tenon, APHP, Paris, France; 10 Service Universitaire des Maladies infectieuses et du Voyageur, Centre Hospitalier de Tourcoing, Tourcoing, France; 11 Service de Médecine interne et Immunologie clinique, Hôpital Bicêtre, APHP, Le Kremlin-Bicêtre, France

**Keywords:** HIV-1, Transmission network, Phylogeography, Primary infection, Treatment as prevention

## Abstract

**Background:**

Characterizing HIV-1 transmission networks can be important in understanding the evolutionary patterns and geospatial spread of the epidemic. We reconstructed the broad molecular epidemiology of HIV from individuals with primary HIV-1 infection (PHI) enrolled in France in the ANRS PRIMO C06 cohort over 15 years.

**Results:**

Sociodemographic, geographic, clinical, biological and *pol* sequence data from 1356 patients were collected between 1999 and 2014. Network analysis was performed to infer genetic relationships, i.e. clusters of transmission, between HIV-1 sequences. Bayesian coalescent-based methods were used to examine the temporal and spatial dynamics of identified clusters from different regions in France. We also evaluated the use of network information to target prevention efforts. Participants were mostly Caucasian (85.9%) and men (86.7%) who reported sex with men (MSM, 71.4%). Overall, 387 individuals (28.5%) were involved in clusters: 156 patients (11.5%) in 78 dyads and 231 participants (17%) in 42 larger clusters (median size: 4, range 3–41). Compared to individuals with single PHI (n = 969), those in clusters were more frequently men (95.9 vs 83%, p < 0.01), MSM (85.8 vs 65.6%, p < 0.01) and infected with CRF02_AG (20.4 vs 13.4%, p < 0.01). Reconstruction of viral migrations across time suggests that Paris area was the major hub of dissemination of both subtype B and CRF02_AG epidemics. By targeting clustering individuals belonging to the identified active transmission network before 2010, 60 of the 143 onward transmissions could have been prevented.

**Conclusion:**

These analyses support the hypothesis of a recent and rapid rise of CRF02_AG within the French HIV-1 epidemic among MSM. Combined with a short turnaround time for sample processing, targeting prevention efforts based on phylogenetic monitoring may be an efficient way to deliver prevention interventions but would require near real time targeted interventions on the identified index cases and their partners.

**Electronic supplementary material:**

The online version of this article (doi:10.1186/s12977-017-0339-4) contains supplementary material, which is available to authorized users.

## Background

Identifying and monitoring HIV-1 transmission networks can be important in understanding the evolutionary patterns and geospatial spread of the epidemic. Recent advances in molecular epidemiology have greatly enhanced our ability to characterize the dynamics and the structure of HIV transmission networks over space and time using HIV-1 *pol* sequences generated for routine [1–3]. A better understanding of the dynamics of the HIV-1 epidemic can assist preventive measures [2–5]. In the past decade, there has been an increase in the circulation of non-B strains and Circulating Recombinant Forms (CRFs) of HIV-1 in Europe and North America. In France and other European countries, HIV-1 subtype B is still predominant but the proportion of non-B infected individuals has progressively increased [6–8]. This increase of non-B viral infections has been reported in both newly diagnosed chronic HIV-1 infections [9, 10] and individuals with primary or recent HIV-1 infection (PHI) [11–14]. Among HIV-1 non-B subtypes, CRF02_AG is one of the most prevalent recombinant forms in the world, responsible for at least 8% of total infections [15]. Though CRF02_AG is predominantly transmitted within heterosexuals in Sub-Saharan Africa, it has been increasingly reported among men who have sex with men (MSM) [16]. In a recent study conducted in newly diagnosed patients living in Europe, the proportion of circulating recombinant form CRF02_AG increased significantly between 2002 and 2010 [17]. A similar trend was also observed in Western and Central Europe, with an increased proportion of CRF02_AG from 5% in 2000–2003 to 8% in 2004–2007 [15]. In France, recent data showed a spread of non-B subtypes in individuals of French origin and that the MSM group are particularly involved in this dynamic [14]. Altogether, these reports emphasize the need for a better understanding of the spread of various HIV-1 subtypes through these transmission networks. The recent advances in molecular epidemiology have greatly enhanced our ability to evaluate the dynamic of these transmission networks [2, 18–21]. Several recent studies have also used clustering approaches to characterize potential correlates of HIV transmission [3, 4, 22] and there is a growing interest in using these approaches to implement and evaluate prevention interventions [1, 2, 23, 24].

In this study, we analyzed HIV-1 *pol* sequences generated over a period of more than 15 years from individuals enrolled during PHI in France in the National Agency for Research on AIDS and hepatitis (ANRS) PRIMO Cohort to reconstruct the broad molecular epidemiology of the HIV epidemic in France. We then determined if clustering analyses could be used efficiently to target prevention interventions in newly diagnosed PHI individuals belonging to an active cluster of transmission (index cases).

## Methods

### Study population

The study protocol was approved by the Paris Cochin Ethics Committee, and all patients gave their written informed consent.

The multicenter ANRS CO6 PRIMO cohort has enrolled in France more than 1900 participants with PHI since November 1996, which were diagnosed on the basis of a negative or incomplete Western blot (no anti-p68 or anti-p34) with detectable HIV-1 RNA for 96% of cases or on the basis of an interval of <3–6 months between a negative and a positive enzyme-linked immunosorbent assay (ELISA) for the remaining cases [25]. All were treatment naïve at enrollment. Demographic, behavioral, biological (CD4, HIV-RNA) and clinical data of the participants were collected and organized anonymously in a common electronic archive.

### HIV-1 *pol* sequencing

For all participants, plasma samples were collected at enrollment in the ANRS-PRIMO cohort, centralized in the Virology Laboratory of Necker Hospital and stored for genotypic resistance testing. HIV RNA was extracted and *pol*-amplified products were sequenced using published primers (http://www.hivfrenchresistance.org, HXB2 coordinates 2530–3334) [6, 26].

### Transmission network

Sequence curation, alignment, and network inference were performed using the freely available software (https://github.com/veg/hivclustering, https://github.com/veg/TN93). After quality control procedures no contaminant sequences were identified [27], and the partial transmission network was inferred based on the nucleotide genetic distances between bulk HIV-1 *pol* sequences from each participant [28].

Similar to previous studies investigating the structure and dynamics of HIV transmission networks [2, 18–21], we linked two individuals (nodes) in networks whenever their *pol* sequences were ≤1.5% distant (TN93 distance measure). This relatively conservative genetic distance cutoff was determined based on previous within host evolutionary rate estimates [29] where HIV sequences from mono-infected participants collected after almost a decade had less than 1% divergence from baseline.

The degree (connectivity) of each individual was defined as the number of links (edges in the transmission network) to other individuals [30]. We also have explored the potential impact of drug resistance mutations (DRM) on network inferences by excluding codons associated with DRM [28, 31]. To prevent spurious sequence linkage due to nucleotide ambiguities, genetic distances between ambiguous nucleotides and known nucleobases were averaged (i.e., R was considered as 50% A and 50% G) [20]. Dyads and clusters were defined as connected components of the network comprising 2 nodes and >2 nodes, respectively. Singletons were defined as individuals without an identified phylogenetic connection.

### Phylogenetic analysis and demographic history

HIV-1 subtypes were determined uploading sequences individually into the REGA HIV-1 automated Subtyping Tool version 2.0 (http://www.bioafrica.net/rega-genotype/html/subtypinghiv.html) and confirmed by in-house phylogenetic analysis.

The age of the most recent common ancestor (TMRCA, years) and the ancestral geographic movements were jointly estimated using a Markov Chain Monte Carlo (MCMC) framework as implemented in BEAST v1.8.1 [32]. We used a discretized gamma distribution (GTR + 4Γ) to account for among-site rate variation. Time scales of the trees were calibrated with the sampling dates available. The temporal scale of evolutionary process was estimated from the sampling dates of the sequences using a relaxed uncorrelated lognormal molecular clock model and a gamma prior on clock rate. Different parametric demographic models (a constant population size, exponential and logistic growth) and a nonparametric Bayesian skyline plot (BSP) were compared, and the best models were selected as confirmed by a higher Bayes Factor (BF) support implemented in BEAST [32]. MCMC simulations were run for 50–200 × 10^6^ chain steps, sub-sampling parameters every 20,000 steps. After removing 10% of burn-in and combining evolutionary parameters and trees using LogCombiner. Convergence of the chains was inspected using Tracer.v.1.5. The TMRCA estimates were expressed as mean and 95% highest posterior density (HPD) years before the most recent sampling dates.

The identification of significant migration pathways was performed using discrete non-reversible diffusion models and a Bayesian stochastic search variable selection (BSSVS) approach [33]. We first applied a discrete diffusion model and geographic locations were recorded at the tips of *pol* phylogenies. To quantify the dissemination process, we estimated the number of viral migrations among locations using ‘Markov Jump’ counts [34] of location-state transitions along the posterior tree distribution [35]. In an attempt to maximize spatial information and put spatiotemporal dynamics in a demographic context, we compiled location information into 10 equally populated areas (regions 1–10). We also included sequences originating from French overseas departments (region 11).

### Statistical analyses

All available data including demographics, HIV risk factor, baseline CD4 count, viral load and HIV-1 subtype or CRF were compared between individuals who clustered and those who did not and analyzed to determine if these factors changed over time. Categorical variables were compared using the Fisher exact test, and continuous variables were evaluated using the Wilcoxon rank test. p values <0.05 were considered statistically significant. The calculations of all statistical tests were performed by using Graph-Pad Prism 6.0c software (GraphPad Software, Inc., San Diego, CA) and the computing R environment.

### Impact of targeted prevention

We evaluated whether phylogenetic monitoring can be used to efficiently target prevention interventions [5]. Here, we evaluated the impact of a prevention intervention targeting clustering individuals (index cases) belonging to an active cluster before 2010 on onward HIV transmission within this network. Briefly, we estimated the number of infections that would have been prevented from 2010 to 2014 if identified clustering individuals (index cases) enrolled in the PRIMO Cohort had received the prevention intervention based on the network density (defined as $\frac{{{\sum }{\text{degrees}}\left( {\text{cluster}} \right)}}{{{\text{size}}\left( {\text{cluster}} \right)}}$) prior to 2010. We then considered that an onward transmission would have been prevented if a participant (contact of index cases) was diagnosed after the date of intervention (i.e. from 2010 to 2014) and belonged to an active transmission cluster targeted by the intervention.

## Results

### Population characteristics

A total of 1356 individuals with PHI enrolled in the ANRS PRIMO cohort CO6 between 1999 and 2014 were included. Participants were preferentially Caucasian (85.9%), male (86.7%) and MSM (71.4%). More than one-third (37%) were diagnosed in Paris (n = 348) and its surrounding areas (n = 167). The median age was 35 years (range from 17 to 79 years), and a vast majority (69%) was between 20 and 40 years old. Over this period, the proportion of HIV-infected individuals reporting MSM risk increased over the study period from 66% (n = 309) in 1999–2005 to 77.7% (n = 310) in 2011–2014 (Chi square test for trend, p < 0.01). The socio-demographic characteristics of 1356 individuals are summarized in Table 1.

**Table 1:** Population characteristics at primary infection

	Not clustered individuals	All clustered individuals	Large clustered individuals (≥3 individuals)	p value^§^
N	71.5% (969)	28.5% (387)	17% (231)	
Age (years) Median (min–max)	36 (17–79)	32.5 (18–68)	32 (18–64)	p < 0.01
Sex				
Male	83.0% (804)	95.9% (371)	98.2% (227)	p < 0.01
Female	16.6% (161)	3.9% (15)	1.3% (3)	
NA	0.4% (4)	0.2% (1)	0.5% (1)	
Ethnicity				
White	83.7% (811)	91.5% (354)	93.1% (215)	p < 0.01
Black	12.8% (124)	5.7% (22)	3% (7)	
Asian	1.5% (15)	1.3% (5)	1.3% (3)	
Others/NA	2.0% (19)	1.6% (6)	2.6% (6)	
Origin				
Paris area^a^	38.4% (372)	37% (143)	41.6% (96)	p = 0.66
Other French regions	55.3% (536)	56.8% (220)	52.8% (122)	
Overseas	6.3% (61)	6.2% (24)	5.6% (13)	
Risk				
MSM	65.6% (636)	85.8% (332)	88.3% (204)	p < 0.01
HTS	27.6% (267)	7.5% (29)	4.3% (10)	
IDU	0.3% (3)	0% (0)	0% (0)	
Others/NA	6.5% (63)	6.7% (26)	7.4% (17)	
Year of diagnosis				
1999–2005	38.4% (372)	24.8% (96)	20.3% (47)	p < 0.01^†^
2006–2010	34.2% (332)	40.6% (157)	43.7% (101)	
2011–2014	27.4% (265)	34.6% (134)	35.9% (83)	
CD4 (cells/µL)—median (IQR)	506 (382–655)	522 (382–655)	525 (379–659)	p = 0.25
HIV-RNA level Log_10_ copies/mL—median (IQR)	5.1 (4.4–5.8)	5.2 (4.7–5.8)	5.2 (4.6–5.8)	p = 0.15
HIV-1 subtype				
B	71.5% (693)	74.9% (290)	69.2% (160)	p = 0.01
CRF02_AG	13.4% (130)	20.4% (79)	28.1% (65)	
A	3.2% (31)	0.2% (2)	0	
C	2.4% (23)	0% (0)	0	
Others	9.5% (92)	3.8% (16)	2.6% (6)	

*MSM* man who have sex with men, *HTS* heterosexual individual, *IDU* injection drug user

^§^Statistical significance was assessed between clustering and non-clustering individuals

^a^Zipcodes: 75, 91, 92,93, 94 and 95; ^†^ Chi square test for trend

### Subtype epidemics

Overall, the HIV-1 subtype B (72.5%, n = 983) was largely predominant. Among the non-B viral strains, CRF02_AG was the most prevalent (56% of non-B, n = 209) (Table 1). MSM individuals were significantly more likely to be infected with subtype B (80%) than CRF02_AG (12.2%) and other non-B lineages (p < 0.001). Among heterosexuals, subtype B clade was also the most prevalent (42%) but prevalence of CRF02_AG (27%) and other non-B subtypes (30.7%) increased overtime. We also showed a significant increase of non-B subtypes among MSM over the study period (p < 0.001).

### Transmission network characteristics

**Fig. 1:**
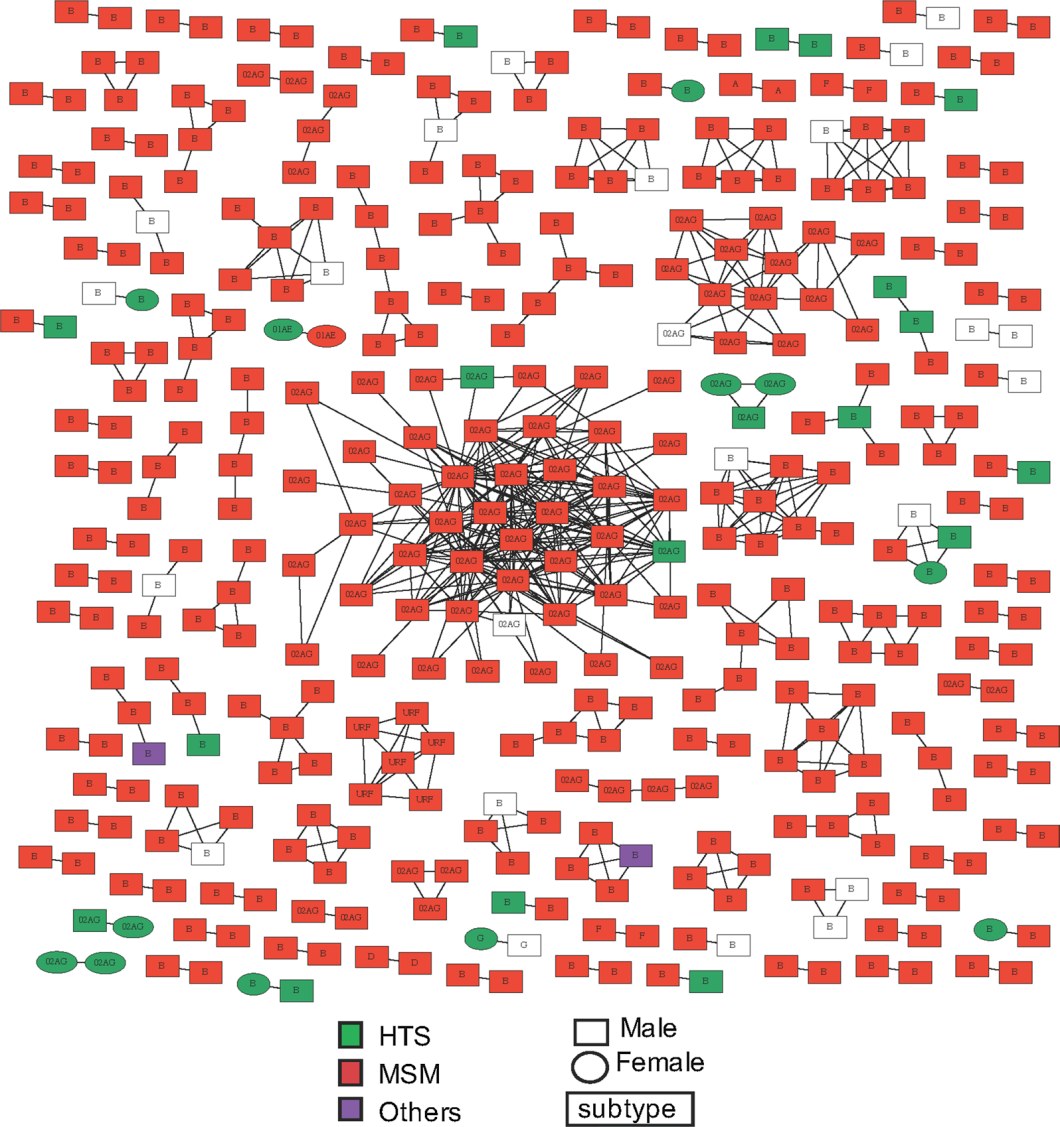
Inferred HIV transmission clusters. HIV-1 transmission cluster diagrams illustrating the structure and demographics of the putative transmission clusters identified in the PRIMO ANRS CO6 cohort. A total of 387 of the 1356 (28.5%) individuals were connected with at least one other individual. *Color* indicates the reported transmission risk [*red* MSM; *green* heterosexual (HTS), *purple* others]; and *shape* denotes gender (*ellipse* male, *square* women). All edges represent a genetic distance of ≤1.5% separating nodes. All *shapes* are labeled according to the HIV-1 subtype. *NA* not available. *White* and *unfilled dots* correspond to missing informations

The HIV-1 *reverse transcriptase* sequences generated from each participant were used to infer the transmission network. Overall, the mean genetic distance between pairs of sequences was 6.5% (s.d. ± 2%) and 5.9% (s.d. ± 2%) among subtype B and CRF02_AG infections, respectively. We used a pairwise distance below the threshold of 1.5% to define a link between individuals [2]. Using this threshold, 387 individuals (28.5%) were connected to at least one other study participant. Exclusion of DRM codons did not impact the observed HIV transmission network. Connected nodes (Fig. 1) were arranged in 44 large clusters (i.e., connected to more than one other individual) ranging in size from 3 to 41 individuals (median = 4) and 78 dyads (i.e., connected with a unique participant). Then, we evaluated the factors associated with clustering. Comparison between people in clusters (i.e. connected) versus people not in cluster (i.e. singletons) revealed that individuals who are linked in a cluster were more likely men (95.9 vs 83%, p < 0.01) and significantly younger (median age = 32.5 vs 36.0 years, p < 0.01) than singletons. MSM (85.8% clustering vs 65.6% non-clustering, p < 0.01) and white participants (91.5 vs 83.7%, p < 0.01) were significantly more likely to cluster. There were no significant associations between clustering and: baseline CD4 T cell count (p = 0.25), baseline viral load (p = 0.15) or being diagnosed in Paris and its suburban area (37.0 vs 38.4%, p = 0.66) (Table 1). Individuals infected with CRF02_AG viruses were also more likely to be connected to another participant than other subtype infections (20.4 vs 13.4%) (Additional file 1: Figure S1). As might be expected, the probability that an individual was connected with another individual increased significantly during the study period (from 1999 to 2014) (p < 0.01).

**Fig. 2:**
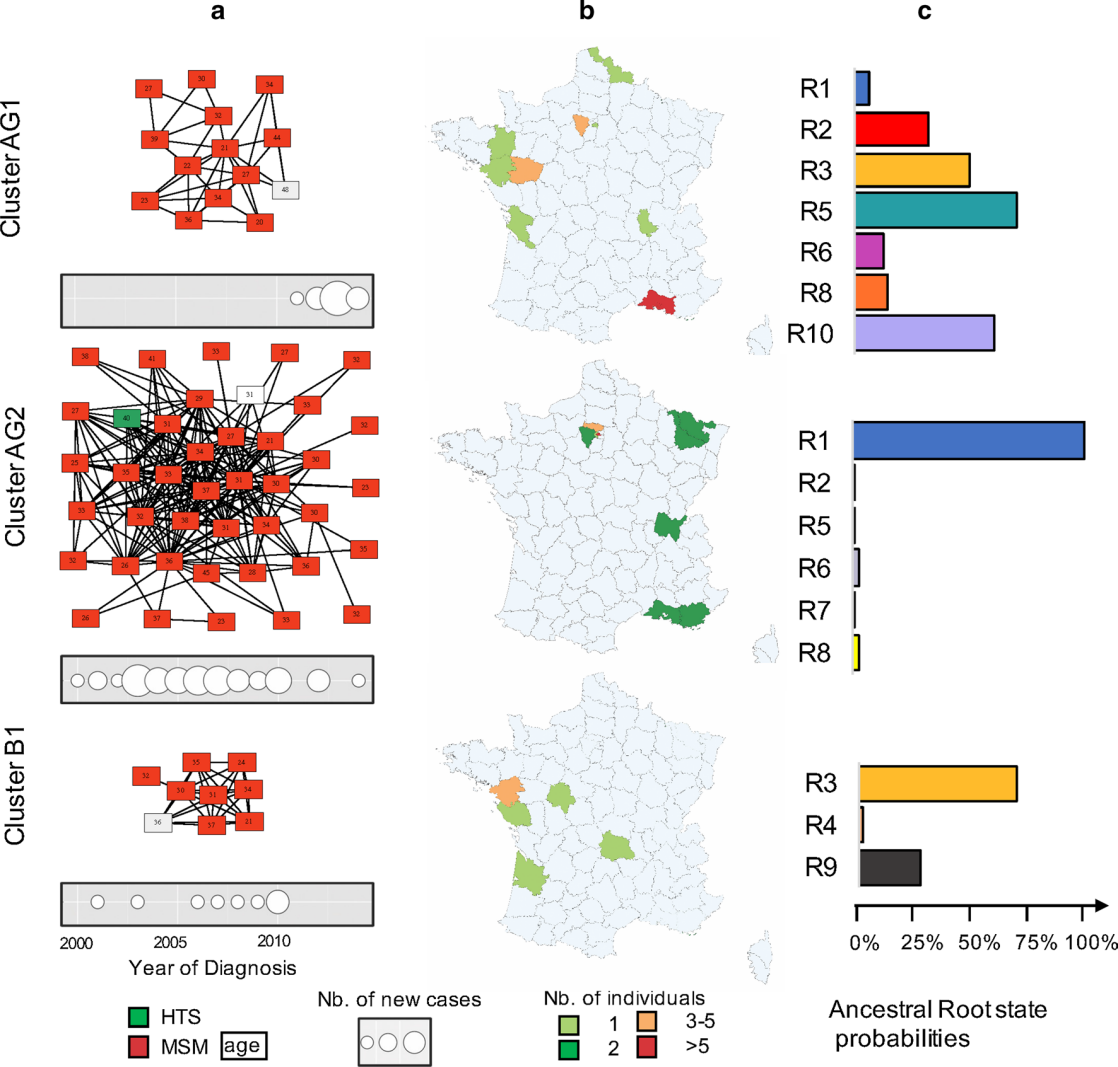
Characteristics of the 3 larger clusters B1, AG1 and AG2. **a** Transmission network of the three larger cluster AG1 (n = 14), AG2 (N = 41) and B1 (n = 9) and evolution of the main clusters over the study period. **b** Map representing the number of clustering individuals by location of residence. **c** Ancestral root state probabilities. The root state probabilities are presented with the *color* codes corresponding to the 11 equally populated regions

**Fig. 3:**
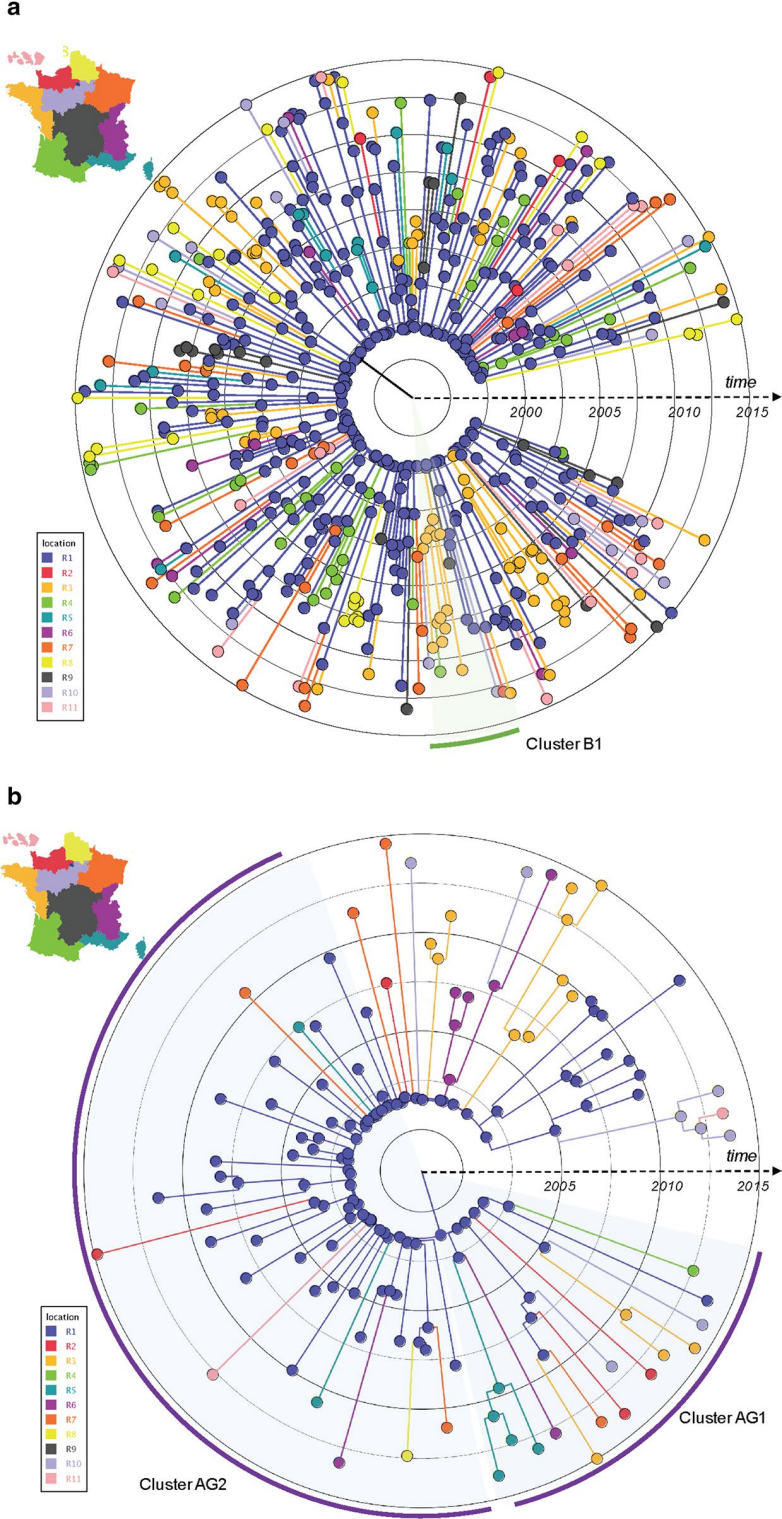
Bayesian time-scaled tree of the HIV transmission network of subtype B (**a**) and CRF02_AG (**b**) *pol* sequences in clusters from the participants enrolled in the PRIMO ANRS cohort between 1999 and 2014. Time scaled in year. Nodes and branches are *colored* according to the most probable location state of their descendent nodes. Tips are *colored* according to the recorded location of sampling

Within the transmission network, three distinctly large viral clusters B1 (subtype B), AG1 and AG2 (CRF02_AG) were identified with a respective size of 9, 14 and 41. Cluster B1 included nine white MSM (median age: 32 years old [21–37]). It was first identified in 2001 with no additional cases since 2010, mostly originating from the Nantes area (West coast) (Fig. 2). Cluster AG1 was composed of fourteen white MSM (median age: 31 years old [20–48]) infected with CRF02_AG-related variants. It was first identified in 2011 and had a peak identification of individuals in 2013. Individuals in this cluster were mostly originating from the Marseille area (South-East) but more widely spread throughout the country (Fig. 2). Finally, cluster AG2 was composed of forty-one white men (median age: 32 years old [21–45]) mostly originating from Paris and its suburban area (n = 34, 83%). It was first noted in 2000 and accrued individuals relatively steadily throughout the observation period (Fig. 2). All but one reported MSM sexual risk exposure. To further evaluate the timing of these clusters, we found that the cluster B1 had a TMRCA in 1997 (95% HPD: 1995–2000) (Fig. 3), and the CRF02_AG clusters AG1 and AG2 had a TMRCA in 2003 (95% HPD: 2000–2008) and 2000 (95% HPD: 1998–2001) respectively (Fig. 3).

### Spatiotemporal patterns of the subtype B and CRF02_AG epidemics

Discrete diffusion models can offer insights into the origins and epidemiological links within the set of locations from which infections were sampled [33, 36].

To analyze the spatial spread of subtype B and CRF02_AG epidemic among individuals with PHI in France between 1999 and 2014 at a national scale, we compiled all subtype B (n = 983) and CRF02_AG (n = 209) sequences. Given the demographic characteristics of France with wide range of population density, we performed our analyzes after dividing the country in 10 equally populated area and an 11th area accounting for individuals originating from overseas departments (n = 86) accounting for 6.3% of the participants.

Geographic locations throughout the phylogenetic histories were estimated by applying a discrete asymmetric Bayesian phylogeographic approach, which allows for possibly different dispersal rates between two locations depending on the directionality of diffusion [37]. Reconstruction of viral migrations across time suggests that Paris area was the major hub of dissemination of both subtype B and CRF02_AG epidemics.

Similarly, we evaluated the dynamics of the three main clusters of subtype B and CRF02_AG lineages. The most probable root location for phylogeny of cluster B1 was the Western region R3 with posterior state probability of 0.72. The most probable root state probabilities were South Eastern regions with posterior state probability and Paris region R1 with posterior state probability of 0.95 for the AG1 and AG2 clusters, respectively (Fig. 2). These results showed that the diffusion of the larger CRF02_AG cluster (AG2) ignited in the most densely populated region (Paris, R1) and thereafter spread to other areas (Fig. 2).

### Targeted ART for prevention

Given the depth of sampling and the characteristics of the ANRS PRIMO transmission network, we evaluated the number of onward transmissions that could have been prevented by an enhanced prevention intervention targeting newly diagnosed clustering HIV-infected individuals (index cases).

We identified that 223 of the 872 (25.6%) newly diagnosed individuals before 2010 in the PRIMO cohort belonged to a transmission network. We estimated that an enhanced prevention intervention on these clustering index individuals could have potentially prevented 60 of the 143 (42%) onward new infections linked to these index cases. The 320 individuals diagnosed between 2010 and 2014 that did not cluster with any individuals infected before 2010 would not have been protected by any prevention intervention given to prior cohort participants. We next evaluated the impact of intervention targeted to participants enrolled before 2010 by cluster densities defined as $\frac{{{\sum }{\text{degrees}}\left( {\text{cluster}} \right)}}{{{\text{size}}\left( {\text{cluster}} \right)}}.$ We found that intensified prevention intervention provided to all individuals belonging to clusters with densities ≥2 (n = 79 individuals) diagnosed before 2010 would have potentially prevented 33 transmissions linked to these index cases.

## Discussion

Here, we investigated the transmission network and spatiotemporal dynamics of HIV-1 epidemic in 1356 primary infected individuals enrolled in the French ANRS PRIMO CO6 cohort between 1999 and 2014. We particularly focused on the major circulating subtypes B and CRF02_AG to provide a better understanding on the dynamics of these epidemics in France and their relative evolution [6, 12, 26, 38–40].

As the epidemic has matured, patterns of HIV transmission have changed, albeit MSM represent a large proportion of the affected populations in many countries [41]. In Western Europe, the incidence of HIV-1 among MSM has increased or at least has remained constant over the last decade [42–44]. The increased proportion of MSM is also observed among all new HIV diagnoses in France (up to 43% in 2013) [45]. Consistent with these observations, we found that the proportion of MSM among all PHI enrolled in the ANRS-PRIMO cohort significantly increased over the last 15 years (from 66% in 1999–2005 to 77.7% in 2011–2014). However, the high frequency of MSM among recently HIV-infected patients can also be partially explained by targeted and repeated screening of MSM populations. For example in the United Kingdom, the proportion of individuals tested for HIV among people attending to STI clinics was the highest among MSM and increased overtime (86% in 2013 vs 78% in 2009) [46]. More recently, a multicenter preventive trial in France (ANRS IPERGAY) also showed a high incidence rate of HIV among MSM, up to 9 per 100 person-years in Paris [47].

By combining methods from classical and molecular epidemiology, we were able to infer and characterize the HIV-1 transmission networks among identified PHI individuals in France over 15 years. Similar to previous data showing that the HIV-1 networks derived mostly from populations of MSM [2, 39, 48], we observed that MSM were significantly more prevalent in connected individuals than singletons. We also showed that non-B subtypes were frequent in primary infected individuals (28.5%), although slightly less than among all new HIV diagnoses in France [10, 45]. We observed that 57% of the individuals infected with CRF02_AG variants reported MSM sexual risk exposure. These results are in line with reports from other European countries showing an increase in proportion of non-B clades in the MSM population [49] suggesting that the sociodemographic boundaries between HIV-1 subtypes globally are diminishing in Western Europe.

By applying Bayesian phylogeographic inference using discrete non-reversible models to *pol* geo-referenced sequences, we also investigated the spatial patterns of subtype B and CRF02_AG clades in France among PHI individuals. We found that these two distinct epidemic lineages have ignited in the most urbanized region of Paris (R1, “Ile de France”) as illustrated by the most probable location state of the descendant nodes with over 90% of all viral lineages movements originating from Paris area (i.e. viral dispersal *from* Paris) for both lineages (96.5% [95% CI 95.1–97.5%] and 94.1% [92.2–95.6%] for subtype B and CRF02_AG respectively). These results suggest the key role of Paris as a hub for new HIV infections and the potential spatial expansion from this region to the rest of the country. This might be explained by the high prevalence of MSM in the ANRS-PRIMO cohort, who are more likely originating from urbanized and highly-densified area [50].

Interestingly, the three larger clusters of HIV-1 subtype B (cluster B1, n = 9) and CRF02_AG (clusters AG1, n = 14 and AG2, n = 41) identified displayed also very distinct evolutionary patterns. While cluster B1 did not increase since 2010, both clusters AG1 and AG2 continued to grow up to end of the follow up period in late 2014. Altogether, these observations suggest that CRF02_AG clade was introduced and disseminated within highly connected networks of MSM, which may explain the successful rapid dissemination and increased prevalence of CRF02_AG subtype [51].

Understanding the dynamic of HIV transmission is crucial in the design of effective interventions and recently Individuals contributes disproportionately to the spread of the HIV epidemic [52–54]. Considering the limited time frame of HIV transmission, targeted prevention strategies focusing on PHI may have a significant impact on the HIV epidemic [52–55]. Here, the extensive collection of samples along with demographic and clinical data of the 1356 PHI participants from the PRIMO ANRS cohort allowed us to better time the observed transmission clusters. It also helped to better evaluate the direct effect of theoretical prevention interventions at an individual level rather than relying on population effect of the intervention (i.e. indirect effect).

Given that the French 2013 guidelines for ART of HIV-1 infection in adults [56] recommend that ART should be initiated in any HIV-positive person, whatever his/her CD4 T cell count, even when >500/mm^3^, we first considered a prevention intervention strategy based on immediate ART introduction [57]. We found that an intensified intervention targeting all clustering participants with PHI diagnosed before 2010 (n = 223 index cases) would have prevented 60 out of the identified 143 onward transmissions identified between 2010 and 2014. Given the depth of sampling and structure of the transmission network, we evaluated intervention strategies based on network connectivity. We found that prevention targeted to newly infected individuals belonging to intermediate and high density clusters (density ≥ 2, n = 79 index cases) would have potentially prevented 33 onward new infections linked to these index cases. In light with a recent study showing that near real-time phylogenetic monitoring of routinely collected HIV genotypes are a promising resource for public health intervention on localized outbreaks of HIV transmission [23], our results also emphasize the need of an efficient sample processing, a rapid turnaround time on HIV sequence generation and a near real time based monitoring of clustering analysis. Beyond the rapid identification of the index cases, an effective and efficient prevention strategy would require a combination of interventions targeting these indices (i.e. immediate provision of ART and enhanced adherence support services), and the identification and immediate provision of pre-exposure prophylaxis (PrEP) to the contacts of these index cases [58]. Identifying and monitoring HIV clusters should be also an invaluable method to (1) determine the leading edge of local HIV transmission, (3) characterize potential correlates of HIV transmission (i.e. recreational drug use, high risk venues) [3, 4, 52], (3) investigate hotspots of transmission [23] and (4) further intensify HIV screening, providing PrEP and partner tracing [59] in population with high rates of transmission.

This study has a number of limitations. First, HIV clustering and network inferences are directly affected by the sampling density [60]: studies with low sampling density showed minimal HIV clustering [61], while in depth sampling allows more accurate characterization of HIV transmission network [48, 49, 62]. In these deeper sampling density studies, the proportion of clustering sequences varied between 28 and 41%, though these studies were performed in heterogeneous population, in other countries and with various methods. While the number of individuals in our dataset is smaller than other studies, we reported an overall clustering proportion of 28.5% and up to 52.5% (332 out of 636) among MSM, consistent with previous reports. This overall clustering rate is also consistent with previous reports where interventions have been deemed useful [5] and allowed the characterization of the dynamics of HIV transmission [19]. We have also estimated that the PRIMO ANRS Cohort is well representative of new HIV infections occurring in France. Indeed, based on the data recorded through the mandatory notification of new HIV infections, 12% of new HIV diagnoses are done at time of primary infection, corresponding to 600–700 cases each year [63]. Therefore, the cases included in the PRIMO ANRS cohort represent approximately 15% of all primary infections diagnosed in the country. In addition, the proportion of MSM included in our study (71.4%) is similar to that observed at the national level (73.6%) [64]. Altogether, while our dataset is not ideal and that we could have missed a number of HIV infected individuals in the local network due to the lack of diagnosis of all PHI, it is sufficient to use the presented techniques to understand the transmission dynamics underlying the sampled epidemic.

Second, recent HIV infection accounts for 39% of new HIV diagnoses in France [45]. Here, we did not take into account the contribution of transmissions from chronically infected individuals to partners diagnosed after the PHI stage, which is also likely to have an important impact on epidemic spread [65] and rely on convenient sampling. Hence, individuals who are not tested or disengaged from care are not represented in this cohort and may also contribute to the epidemic. Though the number of early diagnoses has increased since 2011 in France, the number of late diagnoses has remained stable among MSM, and 25% of the estimated 6220 new HIV diagnoses in 2013 in France were still diagnosed at a late stage (<200 CD4 or AIDS) [45]. Another limitation of all convenience based sampling methods is that the presence of a directed link between two individuals simply reflects recent relatedness of the virus, possibly through a series of unobserved intermediaries [2]. Finally, the use of HIV *pol* region for HIV subtyping may have led to potential misclassification of CRF forms. However, it should not impact the HIV network inferences and the dynamic of CRF02_AG clade within the HIV transmission network presented in this study.

## Conclusion

In summary, this study showed the rapid evolutionary dynamics of subtype B and CRF02_AG among PHI in France since the late 1990s. While subtype B remains the most prevalent lineage, the rapid diffusion of CRF02_AG in highly connected networks of MSM could lead to a substantial and rapid reshaping of the HIV epidemic in Western Countries. Combined with a short turnaround time for sample processing, our findings also show that identifying hotspots of HIV transmission and near real-time monitoring based on phylogenetic analyses can be an effective prevention intervention in combination with Public Health Recommendations for early treatment introduction, enhanced adherence support and partner tracing for immediate diagnosis, provision of PrEP and counseling.

## Additional file

**Additional file 1: Figure S1.** Contingency Plots by Risk (**A**) and by HIV subtype (**B**) among clustering and not clustering individuals. **A** Mosaic plot of the clustering and not clustering individuals according to the risk of HIV acquisition; **B** Mosaic plot of the clustering and not clustering individuals according to the HIV subtype. MSM: Man who have sex with Men; HTS: Heterosexual individual; IDU: Injection Drug User.
