# Supplementary figures and images for: Spatiotemporal dynamics of HIV-1 transmission in France (1999–2014) and impact of targeted prevention strategies
Source: Retrovirology. 2017 Feb 21;14:15. doi: 10.1186/s12977-017-0339-4 (PMC5322782; doi:10.1186/s12977-017-0339-4)

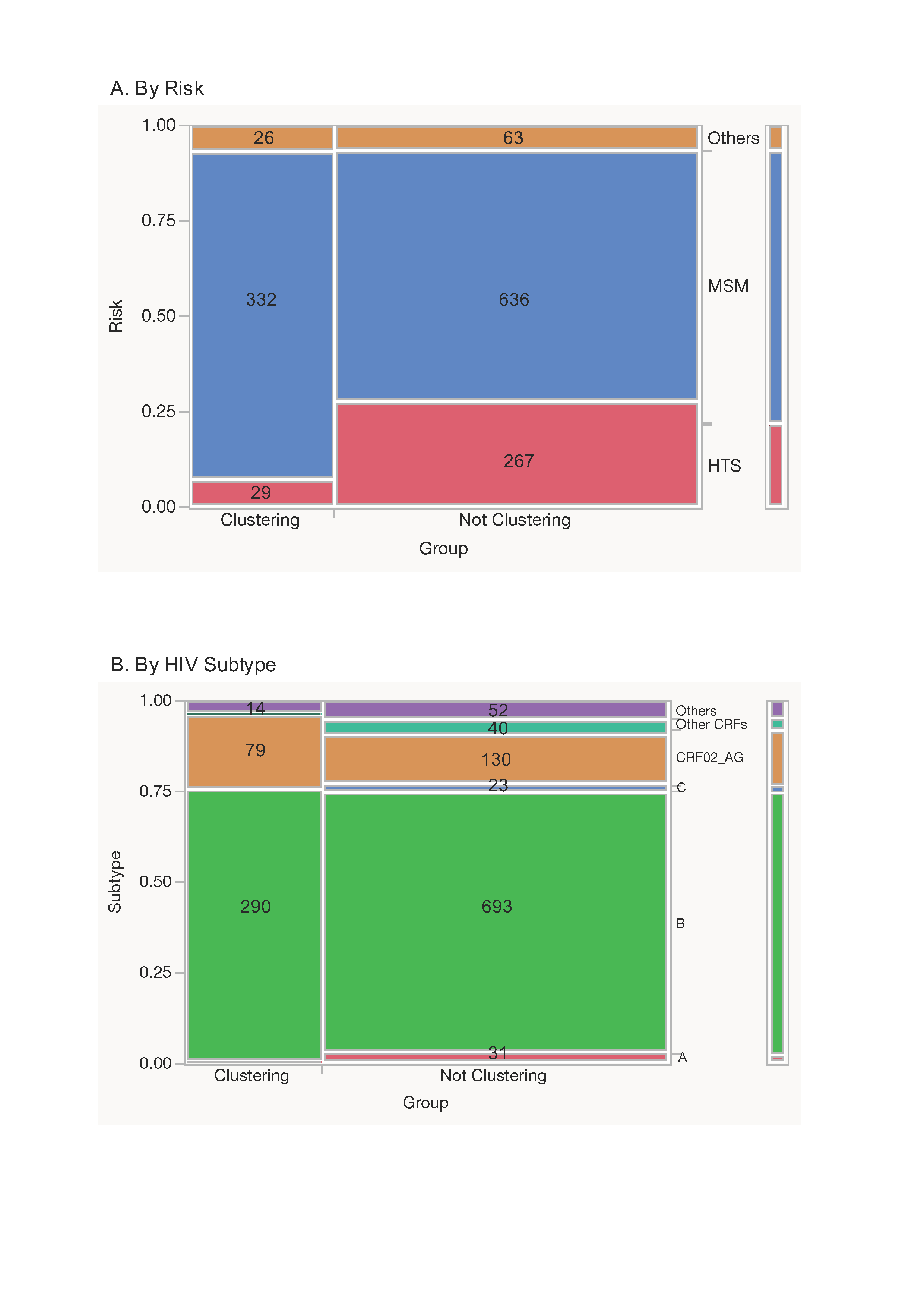

Supplement: Supplementary file 1 — Additional file 1: Figure S1. Contingency Plots by Risk (A) and by HIV subtype (B) among clustering and not clustering individuals. A Mosaic plot of the clustering and not clustering individuals according to the risk of HIV acquisition; B Mosaic plot of the clustering and not clustering individuals according to the HIV subtype. MSM: Man who have sex with Men; HTS: Heterosexual individual; IDU: Injection Drug User. [file 12977_2017_339_MOESM1_ESM.tiff]
